# Development of a novel high-throughput culture system for hypoxic 3D hydrogel cell culture

**DOI:** 10.1038/s41598-024-60822-z

**Published:** 2024-04-30

**Authors:** Dominik Egger, Luisa Baier, Julia Moldaschl, Manfred Taschner, Volker Lorber, Cornelia Kasper

**Affiliations:** 1 Institute of Cell Biology and Biophysics, Leibniz University Hannover, Hannover, Germany; 2 Institute of Cell and Tissue Culture Technologies, Department of Biotechnology, University of Natural Resources and Life Sciences, Vienna, Vienna, Austria; 3 LifeTaq-Analytics GmbH, Tulln an der Donau, Austria

**Keywords:** High-throughput screening, Tissues, Mesenchymal stem cells

## Abstract

Animal models lack physiologic relevance to the human system which results in low clinical translation of results derived from animal testing. Besides spheroids or organoids, hydrogel-based 3D in vitro models are used to mimic the in vivo situation increasing the relevance while reducing animal testing. However, to establish hydrogel-based 3D models in applications such as drug development or personalized medicine, high-throughput culture systems are required. Furthermore, the integration of oxygen-reduced (hypoxic) conditions has become increasingly important to establish more physiologic culture models. Therefore, we developed a platform technology for the high-throughput generation of miniaturized hydrogels for 3D cell culture. The Oli-Up system is based on the shape of a well-plate and allows for the parallel culture of 48 hydrogel samples, each with a volume of 15 µl. As a proof-of-concept, we established a 3D culture of gelatin-methacryloyl (GelMA)-encapsulated mesenchymal stem/stromal cells (MSCs). We used a hypoxia reporter cell line to establish a defined oxygen-reduced environment to precisely trigger cellular responses characteristic of hypoxia in MSCs. In detail, the expression of hypoxia response element (HRE) increased dependent on the oxygen concentration and cell density. Furthermore, MSCs displayed an altered glucose metabolism and increased VEGF secretion upon oxygen-reduction. In conclusion, the Oli-Up system is a platform technology for the high-throughput culture of hydrogel-based 3D models in a defined oxygen environment. As it is amenable for automation, it holds the potential for high-throughput screening applications such as drug development and testing in more physiologic 3D in vitro tissue models.

## Introduction

While animal models continue to hold significance in certain instances, compelling indications suggest the substitution of animal testing. Most importantly, animal models lack physiologic and genetic relevance to the human system which results in low clinical translation of results derived from animal testing^1–3^. Furthermore, besides high costs, animal testing remains morally and ethically questionable^4^. However, the advances in stem cell culture technologies, responsive 3D biomaterials, and automation and monitoring of 3D culture processes have enabled the creation of human 3D in vitro models that more closely mimic the in vivo situation, thus being relevant for drug discovery, disease modeling, and personalized medicine approaches^5^. Moreover, the engineering of personalized in vitro models derived from the patient’s cells holds the potential to enable true personalized diagnosis and treatments^6^.

The 3D culture of cells in hydrogels is the most widely used and relevant culture technique to resemble in vivo-like interactions between cells and the native extracellular matrix (ECM). Although numerous encapsulation techniques and hydrogel compositions are available, methacrylated collagen or gelatin (GelMA) hydrogels are among the most widely used hydrogels and represent an emerging versatile matrix for 3D cell culture^7^. Besides a suitable 3D matrix, other parameters such as the oxygen concentration are crucial to generate an in vivo-like environment. Most cells and tissues reside in an environment with a lower oxygen concentration than the atmospheric concentration of 21% O_2_. Depending on the tissue, the metabolic activity, and the distance to vascularization, the oxygen concentration in the human body ranges from 1% (e.g., in cartilage) to the alveolar oxygen tension of 15% O_2_^8^. The response to reduced oxygen is governed by the group of hypoxia-inducible transcription factors (HIFs)^9^. HIFs are known to directly, mainly by binding to the hypoxia response element (HRE), or indirectly regulate over 1,000 target genes, affecting the energy metabolism, migration, proliferation, differentiation, and immunomodulatory properties amongst others^10,11^. A prominent example of cells that are especially responsive to hypoxia are mesenchymal stem/stromal cells (MSCs) which are one of the most investigated cell types for cell therapy applications^12^ and are used to engineer tissue and disease models or are required in co-culture models due to their physiologic contribution in wound healing^13^, angiogenesis^14^, and immunomodulatory responses^15^. Therefore, for the generation of relevant in vitro models, the characterization and control of the oxygen concentration in 3D is indispensable.

However, to enfold the full potential of 3D in vitro cell constructs for applications such as toxicity screening, biomaterial testing, drug development or drug screening, disease models, culture devices, and automated screening platforms are required that enable the high-throughput culture of 3D models. Examples of platforms for the culture of spheroids or organoids have been described^16–18^. However, only a few approaches exist that allow for the high-throughput culture of hydrogel-based systems^19,20^ which also do not consider the aspect of controlled oxygen levels.

Therefore, in this study, we present a cell culture platform that enables the high-throughput cultivation of 3D hydrogel cultures in a defined hypoxic environment. After the development of the culture platform, we established the culture of GelMA-encapsulated MSCs. As a proof-of-concept, we used the system to generate an oxygen-reduced 3D culture environment and assessed parameters characteristic of oxygen-dependent responses of MSCs.

## Results

### High-throughput 3D cell culture platform

**Figure 1:**
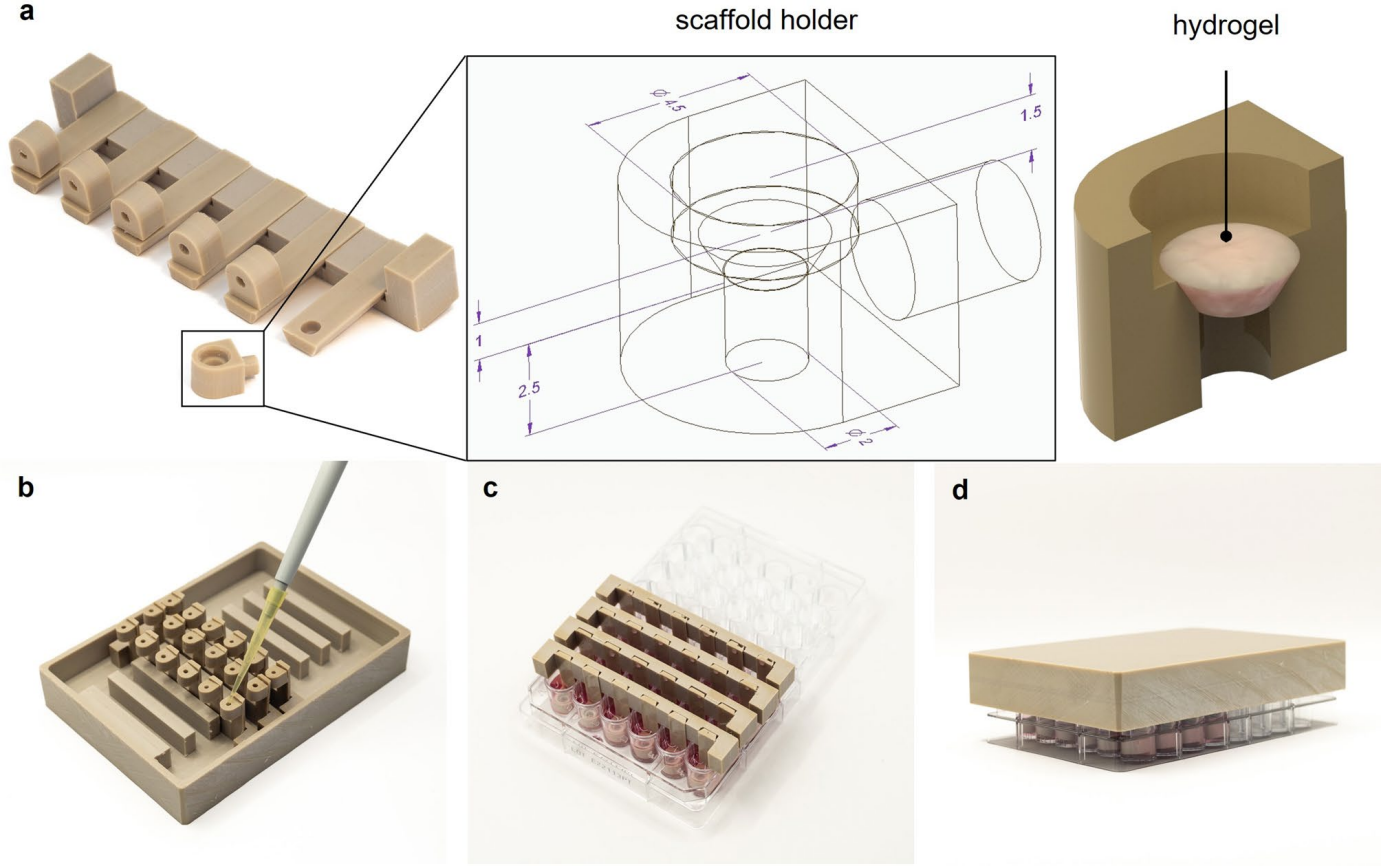
Scheme of the high-throughput cell culture device Oli-Up. The system consists of (**a**) mounts with 6 scaffold holders (dimensions are given in mm). The scaffold holders are manufactured in the shape of a truncated cone which can be loaded with 15 µl hydrogel. (**b**) The scaffold mounts are placed upside down to enable accurate pipetting of the hydrogel into the scaffold holders. (**c**) Up to 8 mounts with each 6 scaffold holders can be placed in a conventional 48-well plate for crosslinking of the hydrogel and addition of medium. (**d**) The Oli-Up system is closed with a modified lid for culture incubation.

In this study, a high-throughput system for the culture of hydrogel-encapsulated cells was developed: the Oli-Up system. It consists of scaffold holders, supporting elements, lids, and autoclave plates (Fig. 1). All parts are made from poly ether ketone (PEEK) ensuring durability and biocompatibility. The scaffold holders can be removed from the supporting element to direct separate samples to offline or endpoint analyses during culture.

**Figure 2:**
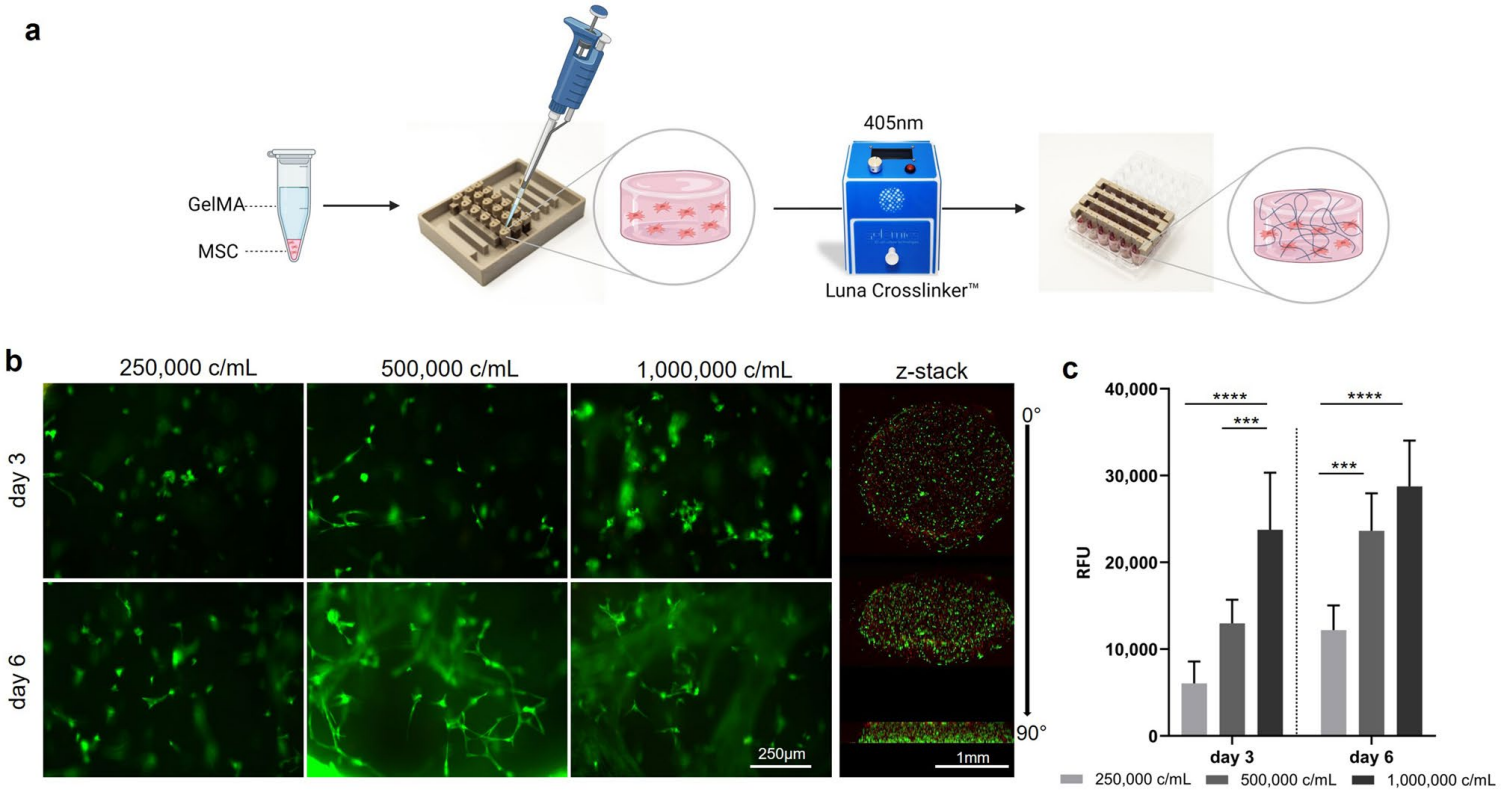
Workflow for the generation of 3D cultures in the Oli-Up system and optimization of the seeding density. (**a**) GelMA-encapsulated MSCs were cast into the scaffold holders of the Oli-Up and photo-crosslinked at 405 nm. (**b**) Representative micrographs and 3D representation of live (green) / dead (red) stain and (**c**) viability assay of GelMA-encapsulated MSCs at different seeding densities and timepoints (n = 3).

To cast the hydrogels, the supporting elements with scaffold holders are placed upside down, 15 µL of a hydrogel-cell solution is dispensed, and crosslinked at 405 nm in the upright position. After adding cell culture medium, the plate is secured with a custom lid and placed in the incubator for further processing (Fig. 2a). To determine the optimal seeding density, MSCs were seeded at 0.25, 0.5, and 1 × 10^6^ c/mL, and the viability was monitored on day 3 and day 6. Between day 3 and day 6, most of the cells developed a spindle-shaped morphology, characteristic of the adherent growing MSCs (Fig. 2b). The response of the viability assay was dependent on the cell number with the highest signal in the highest cell concentration. The viability of cells increased in all cell concentrations from day 3 to day 6 (Fig. 2c).

### Establishing a hypoxic oxygen concentration in the high-throughput 3D culture platform

**Figure 3:**
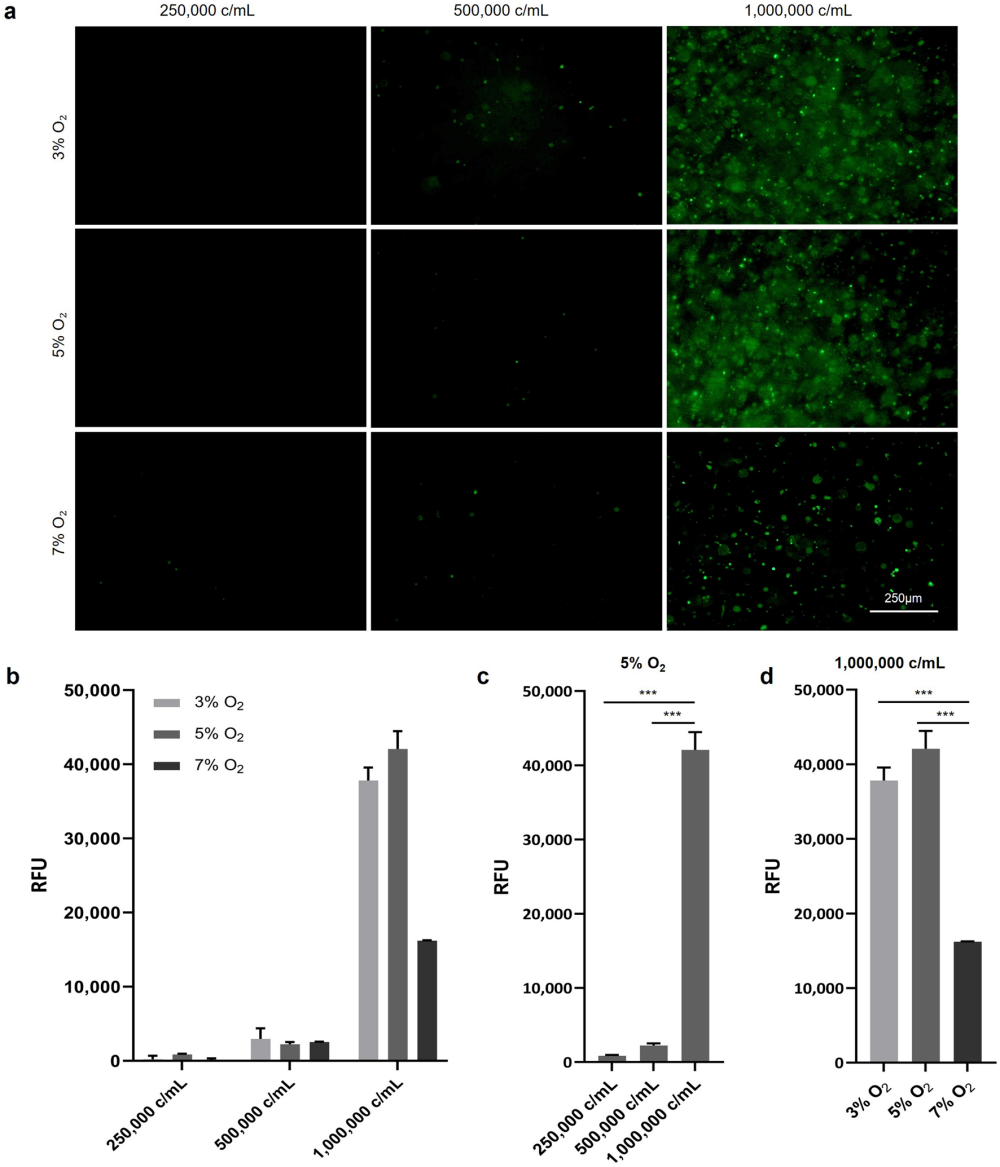
The HRE expression is dependent on the cell density and oxygen concentration. (**a**) Representative micrographs and (**b**) quantitative read-out of the hypoxia-responsive HRE-reporter MSCs encapsulated in GelMA at different seeding densities and oxygen concentrations, measured in a 96-well plate. HRE expression at (**c**) 5% O_2_ and at (**d**) 1 × 10^6^ c/mL reveals that HRE expression is increased at higher cell densities and lower oxygen concentrations (n = 3).

An MSC cell line responsive to the stabilization of HIF-1α was utilized to quantify the response to reduced oxygen concentrations at various cell concentrations. The HRE expression, as indicated by the fluorescent protein UnaG, was significantly elevated in all oxygen concentrations in 1 × 10^6^ c/mL, compared to 0.25 and 0.5 × 10^6^ c/mL (Fig. 3). At 5% O_2_, an approximately 40-fold increase in 1 × 10^6^ c/mL compared to 0.25 × 10^6^ c/mL was observed. Also, at 1 × 10^6^ c/mL a significant oxygen-dependent decrease in HRE expression was observed.

### Oxygen consumption rate of MSCs in GelMA

**Figure 4:**
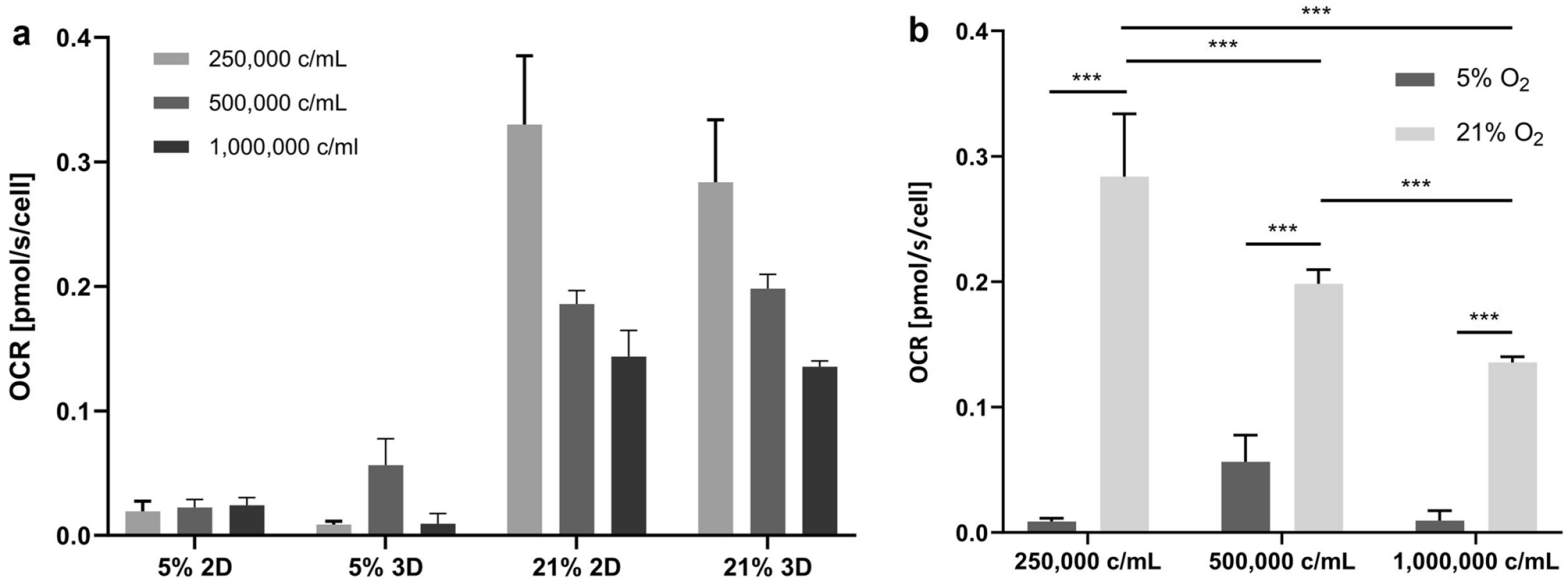
Cell-specific oxygen consumption rate (OCR) of GelMA-encapsulated and 2D MSCs. (**a**) Cell-specific OCR of MSCs in 2D culture and GelMA hydrogels at 21 or 5% O_2_ (n = 3) indicates primarily that OCR levels in both 2D and 3D are highly comparable. (**b**) Cell-specific OCR of MSCs in GelMA at different seeding densities (n = 3) shows that the OCR is dependent on the cell density in normoxia (21% O_2_) but not in hypoxia (5% O_2_) and that the OCR is significantly lower in hypoxic conditions.

The oxygen consumption rate (OCR) of MSCs was determined at 5 and 21% O_2_. Significantly lower OCRs were observed in 5% in all cell concentrations, compared to 21% O_2_ (Fig. 4). While the OCRs were not dependent on the cell concentration in 5% O_2_, a clear decrease in oxygen consumption per cell with increasing cell concentration was observed in 21% O_2_. Anoxia as an indicator of diffusion limitation was not observed in any condition (data not shown).

### MSCs cultured in the Oli-Up system display a hypoxic response on a functional level

**Figure 5:**
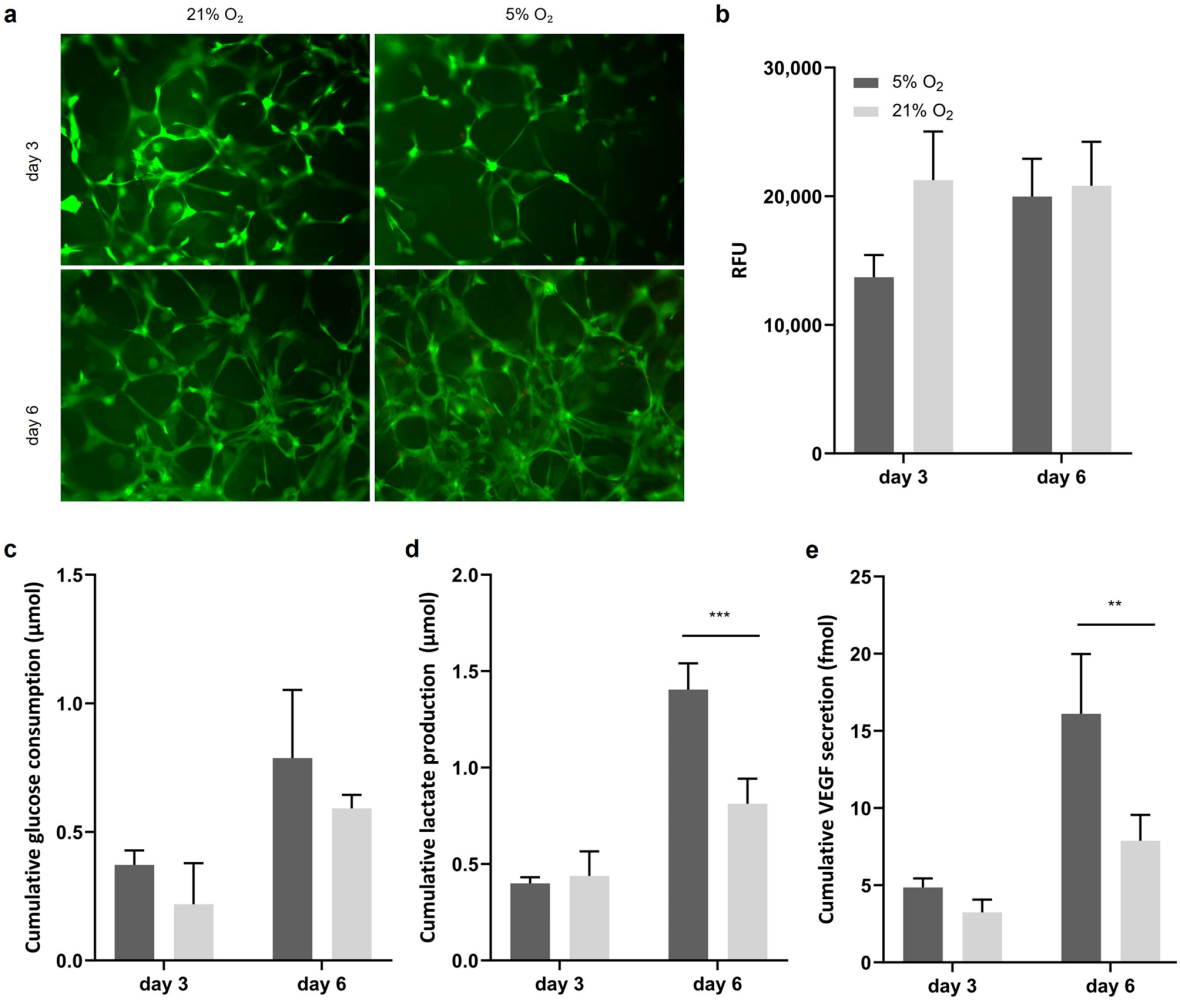
Culture of GelMA-encapsulated MSCs in a defined hypoxic environment. (**a**) Representative micrographs of live (green)/dead (red) stain and (**b**) viability assay of GelMA-encapsulated MSCs at 21 and 5% O_2_ show similar viability in both conditions (n = 3). Characteristic responses to hypoxia were assessed. (**c**) the increased glucose consumption, (**d**) increased lactate production, and (**e**) increased VEGF secretion represent a functional response of MSCs to the hypoxic environment (n = 3).

One aim of this study was to establish a culture environment in which the cells display a clear response to hypoxia. Considering the above findings, we found 1 × 10^6^ c/mL at 5% O_2_ a suitable condition to generate a hypoxic environment. However, we wanted to confirm these findings by observing responses to hypoxia on a functional level. For this, we conducted a 6 day culture in the Oli-Up system and assessed the glucose consumption and lactate production as indicators of a characteristic oxygen-dependent change in the glucose metabolism, as well as the secretion of VEGF which is usually elevated in oxygen-reduced conditions. The viability of cells on day 6 was similar in both conditions (Fig. 5). The glucose consumption was slightly increased while the lactate production was significantly increased in 5% O_2_. The ratio of cumulative lactate production to cumulative glucose consumption, indicative of a change to a hypoxic glucose metabolism, was significantly elevated on day 6 in 5% O_2_ (1.2 ± 0.2 in 21% O_2_ vs. 1.6 ± 0.2 in 5% O_2_). Furthermore, the VEGF secretion was significantly increased in 5% O_2_.

## Discussion

The aim of this study was the development of a cell culture platform that enables the high-throughput cultivation of 3D hydrogel cell cultures in a defined hypoxic environment. The device was required to be scalable, to reduce resources in terms of medium, cells, and hydrogel, to enable medium support from the top and bottom of the hydrogel, to have the flexibility to either generate an air–liquid interface or a submerged culture and the possibility to generate a defined hypoxic environment. To our knowledge, only one similar commercial system is currently available for the high-throughput hydrogel culture. However, the TrueGel3D® HTS Hydrogel Plate (Sigma Aldrich)^21^ is restricted to a specific hydrogel, does not enable the encapsulation of cells into a gel, and supports mass transfer from only one side by covering the hydrogel with medium, similar to a well plate. Also, it does not allow for a potential air–liquid interface. Other custom solutions have been presented. Yu et al. developed a flexible hydrogel-incorporating insert for 24- to 96-well plate^19^. However, the system does also not allow for a potential air–liquid interface. Parish et al. developed a 96-well-based perfused culture platform for screening of tissue-engineered constructs^20^. Although the platform is versatile, it requires complex hardware and is itself not scalable. Considering available systems and custom solutions, the Oli-Up system represents a versatile tool for the high-throughput culture of cells in a variety of hydrogels, applicable to diverse research areas.

As a proof-of-concept, we used the Oli-Up to establish a tightly controlled oxygen-reduced culture environment to induce a functional hypoxic response in GelMA-encapsulated MSCs. Using a hypoxia reporter cell line, we found the expression of HRE was dependent on the cell concentration rather than on the oxygen concentration. For example, a 40-fold increase of cell-specific HRE expression was observed in 1 × 10^6^ c/mL compared to 0.25 × 10^6^ c/mL. Assuming a similar cell number as initially seeded, this would correspond to a tenfold increase in cell-specific HRE expression. We assume that the increased HRE expression in high cell concentration is not caused by diffusion limitation but by an interplay of increased cell–cell contacts and oxygen deprivation. As the expression declined in 3%, compared to 5% O_2_, we defined a cell concentration of 1 × 10^6^ c/mL at 5% O_2_ as the optimal condition to generate a hypoxic environment. However, it was not clear if anoxia due to oxygen diffusion limitation in the GelMA constructs occurred. Thus, we monitored oxygen and assessed the OCR of MSCs in 2D and the GelMA constructs in 5 vs. 21% O_2_. The cell-specific O_2_ consumption at 21% O_2_ in 2D and 3D was clearly dependent on the oxygen concentration with the lowest OCR at the highest cell concentration. This phenomenon is known among different cell types and has been attributed to the fact that a high-density culture better resembles the in vivo situation. At high density culture or in vivo cells are assumed to be less metabolically “stressed”^22^ which is supported by the fact that cells display lower OCRs in vivo than in vitro ^23–25^. However, the cell density-dependent OCR reduction was not observed in hypoxia. Overall, the OCR in hypoxia was significantly lower compared to 21% O_2_ (0.25 × 10^6^ c/mL 29-fold ± 5; 0.5 × 10^6^ c/mL threefold ± 1; 1 × 10^6^ c/mL 25-fold ± 17). It is well known that MSCs reduce mitochondrial oxygen consumption together with energy metabolism^26^. Thus, the cell-density-dependent OCR reduction might not occur in hypoxia as the oxygen consumption itself is altered and reduced to a minimum. The OCRs in 2D and 3D were similar and anoxic conditions were not recorded during the oxygen measurements (data not shown). Together with the findings above, we conclude that a hypoxic environment was established avoiding diffusion limitation and resulting in anoxic region GelMA constructs of the Oli-Up system.

Besides the expression of HRE which is indicative of a hypoxic response of MSCs, we wanted to assess the hypoxia response on a functional level. Therefore, we measured the glucose metabolism and VEGF secretion which are indicative of a hypoxic response in MSCs in a 6-day culture. As the response of the viability assay did not change significantly during the culture, we assume that the cell number was similar on day 3 and day 6 as this was also observed at 1 × 10^6^ c/mL in the previous culture (Fig. 2a and b). The considerably elevated glucose consumption and significantly elevated lactate production are characteristic of a switch in the energy metabolism due to HRE expression^27^. MSCs shift the ATP production from oxidative phosphorylation to glycolysis increasing the consumption of glucose and production of lactate. Especially the ratio of lactate to glucose, which, was significantly elevated, mirrors this shift. Similarly, the VEGF secretion as a target gene of HIF-1α was significantly increased under hypoxic conditions. Taken together we demonstrated the potential of the high-throughput culture platform to establish an environment that was tightly controlled to trigger hypoxia-related responses in MSCs.

Finally, we would like to discuss the limitations of our study. While this proof-of-concept study shows the potential of the developed platform technology it is important to mention that it was not possible to reliably determine the exact cell number during each timepoint of the cultures. Furthermore, the OCR of MSCs could not be determined directly in the Oli-Up system. However, we kept the differences to a minimum by keeping important parameters (distance to medium surface, volume of the hydrogel, cell density) identical. Furthermore, we would like to highlight, that the potential of the presented platform exceeds the presented application of establishing a hypoxic environment by using other cells, hydrogels, and read-outs.

In this study, we developed a high-throughput culture platform for hydrogel cultures and presented a proof-of-concept study with GelMA-encapsulated MSCs in a controlled hypoxic environment. While establishing a controlled hypoxic environment for 3D hydrogel cultures is a valuable application of the Oli-Up platform, its capabilities extend far beyond this specific use case as hydrogels and cells can be adjusted to the researcher's needs, allowing for the generation of various tissue and disease models in the future. The platform also allows for the potential measurement of various cellular parameters, making it a versatile tool for diverse cell culture studies. Furthermore, the system is planned for integration into a completely automated cell culture facility which is currently in development. Here, magnetic fields in combination with permanent magnets act as transport vehicles to transfer cells from incubators to several stations for liquid handling and exchange (e.g., for media change or quality control). Combined with the cultivation device, the system will provide more standardized and reproducible models for efficient drug development mainly based on automated in vitro 3D cell cultivation. The translation to the fully automated facility will enable scalable high-throughput screening of hydrogel-based 3D in vitro models. Furthermore, the Oli-Up system is designed to also host in vitro tissue and or disease models that require an air–liquid interface, such as lung epithelium or skin equivalents. Therefore, future directions will explore the platform’s potential to develop various tissue and disease models for high-throughput applications in drug screening or personalized medicine approaches. With plans for integration into an automated cell culture facility underway, the Oli-Up system promises scalable high-throughput screening capabilities for hydrogel-based 3D in vitro models.

## Materials and methods

### Development of the oli-up system

The Oli-Up System was engineered as a reusable system, avoiding the necessity for component replacement. Individual components were crafted utilizing commercial CAD software (Solid Edge 2023, Siemens PLM Software, Plano, TX, USA). Initial design prototypes were fabricated employing an FDM-3D Printer (Dimension Elite, Stratasys, Rechovot, Israel). Following a comprehensive reassessment of dimensions and configurations, *.dxf files were generated for CNC-cutting, with subsequent preparation of G-Code facilitated through VCarve Pro (Vectric Ltd, Alcester, UK). This G-Code was subsequently utilized for the precise cutting of molding blanks composed of PEEK (GM GmbH, Munich, Germany) on a CNC machine (PFK 1607 PX, BZT Maschinenbau GmbH, Leopoldshöhe, Germany).

### Cell culture

The use of human tissue from an abdominoplasty was approved by the ethics committee of the Medical University Vienna, Austria (EK Nr. 957/2011, 30 January 2013). All experiments were performed in accordance with relevant guidelines and regulations. Informed written consent was obtained from the donor (female, 28 years old). Human MSCs were isolated within 8 h after surgery as previously described^28^. MSCs were cultivated in standard culture medium composed of MEM alpha (Thermo Fisher Scientific, Waltham, MA, USA), 0.5% gentamycin (Lonza, Basel, Switzerland), 2.5% human platelet lysate (hPL; PL BioScience, Aachen, Germany; filtered through 0.2 μm filters) and 1 IU/mL heparin (Ratiopharm, Ulm, Germany) in a humidified atmosphere at 37 °C, 5% CO_2_ and 21% O_2_, and cryo-preserved in liquid nitrogen. Upon use, cryopreserved MSCs were thawed and subcultivated to passage 2. Cell detachment was performed by accutase (Sigma Aldrich, St. Louis, MO, USA) treatment. For subcultivation, cells were seeded at 3000 cells/cm^2^ and allowed to grow 48 h before harvest.

### 3D cell culture in the oli-up system

All components of the Oli-Up system were sterilized by steam sterilization for 20 min at 121 °C in an autoclave (Varioklav 500E, Thermo Scientific). For culture in the Oli-Up system, MSCs were embedded in Lunagel (Gelomics, Brisbane, Australia) a photo-crosslinkable gelatin-methacryloyl (GelMA) hydrogel, at 0.25, 0.5, or 1 × 10^6^ cells/mL. The GelMA was freshly prepared by mixing prewarmed gelatin and the photoinitiator according to the manufacturer’s instructions.

For the seeding process, the supporting elements with attached scaffold holders were placed upside down and the scaffold holders were filled with 15 µL of the cell suspension. Next, the supporting elements were transferred to a 48-well plate (Greiner Bio-One, Frickenhausen, Germany) and the GelMA was crosslinked for 2 min at 405 nm and 9 mW/cm^2^, resulting in a stiffness of 3.5 kPa, according to the manufacturer’s protocol. This stiffness was chosen, as it is supposed to reflect the stiffness of adipose tissue and thus the native environment of adMSCs^29^. Subsequently, 600 µL cell culture medium was added to each well and the plate was placed in a humidified incubator at 37 °C, 5% CO_2_, and the respective oxygen concentrations ranging from 3 to 21% O_2_. MSCs were cultured for 24 h for determination of the OCR or 6 days for the other experiments. Medium samples of 500 µL were collected on days 3 and 6 of the culture and stored at -20 °C for further analyses.

### Hypoxia-responsive reporter cell line

A hypoxia-responsive MSC reporter cell line (HRE-MSC) with a genetically encoded hypoxia sensor was established before^30^ and used to determine the cellular response to an oxygen-reduced environment. In this cell line, the transcription factor hypoxia-response-element (HRE) which is activated upon HIF-1α stabilization is coupled to the expression of dUnaG, a bilirubin-dependent fluorescent protein. As hPL which was used in the culture of the primary MSCs does not contain bilirubin, the medium for the HRE-MSC was supplemented with 10% human serum (Sigma Aldrich) instead of 2.5% hPL. Detection of the fluorescence signal was not possible in the Oli-Up system, hence the measurements were performed in a 96-well plate. For this, HRE-MSCs were embedded at 0.25, 0.5, or 1 × 10^6^ cells/mL in 100 µl GelMA (n = 3), covered with the respective medium and incubated at 37 °C, 5% CO_2_ and 3, 5 or 7% O_2_. After 72 h of cultivation, the cells were observed by fluorescence microscopy and the signal was quantified using the Infinite M1000 plate reader (Tecan, Männedorf, Switzerland) measuring the fluorescence intensity at 498nm_ex_/527nm_em_.

### Oxygen consumption rate

The oxygen consumption rate (OCR) of MSCs encapsulated in GelMA (3D) at 5 and 21% O_2_ was determined by the optical read-out with (VisiSens camera by PreSens, Regensburg, Germany) of sensor spots (PreSens) integrated in a 48-well plate. To generate similar conditions as in the Oli-Up system, a droplet with 15 µL GelMA cell suspension with the same cell concentration as used in the Oli-Up system was pipetted directly onto each sensor spot, followed by crosslinking as described above. A 2D culture with the same absolute cell number as used in the Oli-Up system served as control. The cell-to-air distance was set to match the Oli-Up system by adjusting the medium volume. Overall, n = 3 technical replicates per condition were measured. The oxygen concentration was monitored by an optical readout with the VisiSens camera.

The OCR (pmol s^-1^) was then calculated using the equation

$$OCR=\frac{-DS\alpha \left[p{O}_{2}\left(h\right)-p{O}_{2}\left(0\right)\right]}{h}$$

where *D* is the diffusion coefficient of O_2_ in water, *S* the area of oxygen exchange, α the solubility coefficient at 37 °C, *h* the height of the medium column (cm), *pO*_2_(*h*) partial pressure of oxygen at the level of the cell layer, and *pO*_2_(*0*) the partial pressure of oxygen at the surface of the medium.

The pO2(h) 24 h post-seeding was used to ensure that the majority of the cells were attached but not started to proliferate yet. To calculate the oxygen utilization per cell (pmol s^-1^ cell^-1^), OCR was divided by the cell number.

### Determination of glucose, lactate, and VEGF

Cell culture supernatants were collected for the calculation of glucose consumption, lactate production, and VEGF secretion as described above (n = 3 per condition). The samples were thawed overnight at 4 °C and either measured with the YSI 2900 Biochemistry Analyzer (YSI, Yellow Springs, OH, USA) for determination of glucose and lactate or the concentration of VEGF was determined via Enzyme-Linked Immunosorbent Assay (ELISA, Biotechne, R&D systems, Minneapolis, MN, USA). Cell culture medium which was incubated in the Oli-Up but without cells, served as control. The glucose consumption, lactate production, and VEGF secretion were calculated from this data, respectively. The presented values are blanked and normalized to day 0.

### Metabolic activity

To measure the metabolic activity of cells in the GelMA hydrogels, a resazurin-based viability assay was used (In Vitro Toxicology Assay Kit, TOX8, Sigma Aldrich). The scaffold holders with the hydrogels were transferred to the wells of a 48-well plate and covered with 500 µl cell culture medium supplemented with 10% TOX8 solution. The samples were incubated for 3 h at 37 °C, 5% CO_2_, and 21 or 5% O_2_, respectively, on a horizontal shaker (n = 3 replicates per condition). 200 µL of the supernatant was transferred to a 96-well plate and fluorescence intensity at 560 nm_ex_/590 nm_em_ was measured using the Infinite M1000 plate reader (Tecan, Männedorf, Switzerland). Cell culture medium which was incubated in the Oli-Up but without cells, served as control. The presented values are blanked and normalized to day 3.

### Live/dead staining

To stain viable and dead cells in the GelMA hydrogels, the scaffold holders with the hydrogels were transferred to a new well, covered with a solution of 1 μg/mL calcein AM and 3.3 μg/mL propidium iodide (PI, both Thermo Fisher Scientific) in cell culture medium and incubated for 1 h at 37 °C and 5% CO_2_ on a horizontal shaker in the dark. After washing of the samples with prewarmed medium, the samples were analyzed by fluorescence microscopy (Leica DM IL LED) or scanning confocal microscopy (Leica TCS SP8-STED). Confocal images were processed with FIJI (ImageJ) and the 3D reconstruction was generated using the “3D Viewer” plug-in.

### Statistical analysis

All quantitative data are presented as mean ± the standard deviation (SD) with at least three independent replicates and the sample size “n” of the experiment is given in the legend of each corresponding figure. For multiple comparisons, two-way ANOVA followed by Tukey’s multiple comparisons test was performed with a confidence interval of α = 0.05, and data were plotted using GraphPad Prism 9.0.0 for Windows (GraphPad Software, San Diego, CA, USA). Significance is indicated as follows: *p < 0.05, **p < 0.01, ***p < 0.001, ****p < 0.0001.

## Data Availability

The datasets generated during and/or analyzed during the current study are available from the corresponding author on reasonable request.
